# Management of spontaneous pneumothorax in patients with COVID-19

**DOI:** 10.1093/icvts/ivab280

**Published:** 2021-10-18

**Authors:** Hakki Ulutas, Muhammet Reha Celik, Ilham Gulcek, Muhammed Kalkan, Mehmet Agar, Talat Kilic, Emine Gulcek

**Affiliations:** Department of Thoracic Surgery, Faculty of Medicine, University of Inonu, Malatya, Turkey; Department of Thoracic Surgery, Faculty of Medicine, University of Inonu, Malatya, Turkey; Department of Thoracic Surgery, Faculty of Medicine, University of Inonu, Malatya, Turkey; Department of Thoracic Surgery, Faculty of Medicine, University of Inonu, Malatya, Turkey; Department of Thoracic Surgery, Faculty of Medicine, University of Inonu, Malatya, Turkey; Department of Pulmonology, Faculty of Medicine, University of Inonu, Malatya, Turkey; Department of Pulmonology, Faculty of Medicine, University of Inonu, Malatya, Turkey

**Keywords:** Spontaneous pneumothorax, Coronavirus disease 2019 pneumonia, Chest tube thoracostomy, Ground-glass opacities

## Abstract

**OBJECTIVES:**

The coronavirus disease 2019 (COVID-19) pneumonia may cause cystic features of lung parenchyma which can resolve or progress to larger blebs. Pneumothorax was more likely in patients with neutrophilia, severe lung injury and a prolonged clinical course. The timely diagnosis and management will reduce COVID-19-associated morbidity and mortality.

**METHODS:**

We present 11 cases of spontaneous pneumothorax managed with chest tube thoracostomy or high-dose oxygen therapy. Isolated spontaneous pneumothorax was detected in all cases.

**RESULTS:**

Eight cases were male and 3 cases were female. There were bilateral ground-glass opacities or pulmonary infiltrates in the parenchyma of the 10 cases. We detected neutrophilia, lymphopaenia and increased C-reactive protein, Ferritin, lactate dehydrogenase, D-Dimer, interleukin-6 levels in almost all cases. Chest tube thoracostomy was sufficient to treat pneumothorax in our 9 of case. In 2 cases, pneumothorax healed with high-dose oxygen therapy. Favipiravir and antibiotic treatment were given to different 10 patients. In our institution, all patients with COVID-19 infection were placed on prophylactic or therapeutic anticoagulation, unless contraindicated. The treatments of patients diagnosed with secondary spontaneous pneumothorax during the pandemic period and those diagnosed with secondary spontaneous pneumothorax in the previous 3 years were compared with the durations of tube thoracostomy performed in both groups.

**CONCLUSIONS:**

The increased number of cases of pneumothorax suggests that pneumothorax may be a complication of COVID-19 infection. During medical treatment of COVID-19, pneumothorax may be the only reason for hospitalization. Although tube thoracostomy is a sufficient treatment option in most cases, clinicians should be aware of the difficulties that may arise in diagnosis and treatment.

**Figure:**
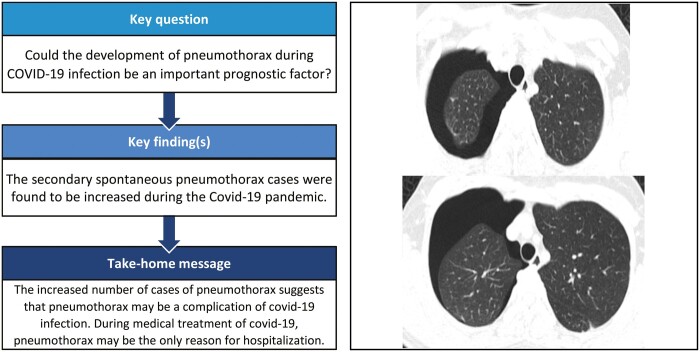

## INTRODUCTION

Coronavirus disease 2019 (COVID-19) pneumonia may cause cystic features in the lung parenchyma, which can resolve or progress to larger blebs [1, 2]. This can place patients at risk of rupture resulting in mediastinal and subcutaneous emphysema or secondary spontaneous pneumothorax (SSP). While there is no known cause of primary spontaneous pneumothorax, SSP may develop in the presence of an underlying lung disease. Spontaneous pneumothorax (SP) has been reported as a complication of COVID-19 with published incidences of 1% in hospitalized patients [3], 3% in patients hospitalized with pneumonia [4], 6% in mechanically ventilated patients [5] and 1% in deceased patients [6]. Due to limited knowledge of lung histopathology in patients with COVID-19, it is unclear how well the diseased lung tissue will spontaneously heal and re-expand without intervention. There have been descriptions of how viral dissemination might be contained using bespoke viral filtration systems to limit contamination [7]. Pneumothorax was more likely in patients with neutrophilia, severe lung injury and a prolonged clinical course. Similarly, pneumothorax has been noted as a poor prognostic feature of Middle East respiratory syndrome coronavirus (MERS)-related infection [8]. Timely diagnosis and management will reduce COVID-19-associated morbidity and mortality.

## MATERIALS AND METHODS

Here, we report the management of 11 cases of SSP detected among COVID-19 patients in our hospital between March 2020 and November 2020. Patients in whom the pneumothorax developed as a consequence of trauma, iatrogenic trauma, barotrauma or other secondary causes were excluded. COVID-19 diagnoses were confirmed using the reverse transcription-polymerase chain reaction technique based on severe acute respiratory syndrome coronavirus 2 antigen detection in nasopharyngeal swabs. The treatments of patients diagnosed with SSP during the pandemic period and those diagnosed with SSP in the previous 3 years were compared and the durations of tube thoracostomy performed in both groups, as well.

All analyses were performed using the Mann–Whitney non-parametric test if non-normally distributed (*P* < 0.05). The demographic, clinical, laboratory and imaging data of those patients who developed an SP are shown in Tables 1 and 2.

**Table 1:** Demographic, clinical and imaging data of patients

Variables	Case 1	Case 2	Case 3	Case 4	Case 5	Case 6	Case 7	Case 8	Case 9	Case 10	Case 11
Age	40	47	75	70	79	86	76	72	88	91	69
Sex	Female	Male	Male	Female	Male	Male	Female	Male	Male	Male	Male
Symptoms	Dyspnoea, sore throat	Dyspnoea, palpitation	Dyspnoea, chest pain, cough and sputum.	Dyspnoea, cough	Dyspnoea	Dyspnoea, fever and weakness.	Dyspnoea	Chest pain	Loss of appetite, Weakness	Dyspnoea	Dyspnoea
Duration of symptoms (day)	3	4	10	4	7	6	4	4	15	2	1
Smoking	Non-smoker	Smoker	Ex-smoker	Non-smoker	Smoker	Ex-smoker	Non-smoker	Ex-smoker	Non-smoker	Smoker	Non-smoker
Lung disease history	No lung disease	COPD	No lung disease	No lung disease	No lung disease	No lung disease	No lung disease	No lung disease	No lung disease	COPD	No lung disease
Pneumothorax	Right	Right	Left	Left	Right	Right	Right	Left	Left	Right	Right
Associated radiological findings	None	Bilateral ground-glass opacities	Bilateral ground-glass opacities pleural effusion, cystic cavitary lesions	Bilateral ground-glass opacities	Bilateral ground-glass opacities	Bilateral ground-glass opacities, pleural effusion	Bilateral ground-glass opacities	Bilateral ground-glass opacities	Bilateral ground-glass opacities	Bilateral emphysema and pulmonary infiltrations	Bilateral pulmonary infiltrates
O_2_ saturation,	80% on room air	56% on room air	75% on room air	80% on room air	50% on room air	70% on room air	85% on room air	92% on room air	80% on room air	88% on room air	90% on room air
O_2_ support at time of pneumothorax	90% on 5 lt/min O_2_	80% on 5 lt/min O_2_	82% on 4 lt/min O_2_	89% on 5 lt/min O_2_	60% on 5 lt/min O_2_	85% on 4 lt/min O_2_	90% on 4 lt/min O_2_	98% on 5 lt/min O_2_	90% on 2 lt/min O_2_	91% on 2 lt/min O_2_	96% on 2 lt/min O_2_
CTT time (days)	5	6	11	6	2	2	3	3	–	–	5
Ventilator required	Not required	NIMV	MV	Not required	NIMV- MV	NIMV	NIMV	Not required	Not required	Not required	Not required
Patient outcome	Discharge	Discharge	Exitus	Discharge	Exitus	Exitus	Discharge	Discharge	Discharge	Discharge	Discharge
Length of stay in the hospital	6	20	11	7	6	4	25	5	13	7	6
Treatment	Favipiravir	Favipiravir Meropenem Tigesiklin	Piperacillin-Tazobactam Meropenem	Favipiravir Sulbactam-Ampicillin Ciprofloxacin	Favipiravir Piperacillic-Tazobactam	Favipiravir Piperacillic-Tazobactam	Favipiravir Sulbactam-Ampicillin	No drug treatment	Favipiravir Piperacillic-Tazobactam	Favipiravir Metilprednisolon Meropenem Clarithromycin	Favipiravir

COPD: chronic obstructive pulmonary disease; CTT: chest tube thoracostomy; MV: mechanical ventilation; NIMV: non-invasive mechanical ventilation.

**Table 2:** Laboratory findings of patients

Laboratory findings		Case 1	Case 2	Case 3	Case 4	Case 5		Case 6	Case 7	Case 8	Case 9	Case 10	Case 11
Neutrophil (10³/µL) (2.1–6.1)	Number	7.45	7.34	10.16	1.49	11.38		17,59	6,14	6,91	7.53	6.51	6.36
Percentage (41–73%)	(%)	83.7	74.6	78.1	55.6	92.1		75.9	92.5	92.1	87.9	92.8	78.3
Lymphocyte (10³/µL) (1.3–3.5)	Number	0.93	1.98	1	1.01	0.14		10.5	0,21	0,33	0.31	0.41	0.79
Percentage (%19.4–44.9)	(%)	10.4	19.1	8.5	37.7	1.1		36.7	3.2	6.2	3.6	5.8	12.2
**CRP (mg/L) (0–0.35)**			1.91	6.17	6.03	5.93		14	37	3,05	12.9	1.04	1.03
**Ferritin (ng/mL) (22–322)**			756.2	>1650	>1650	265		880,5	>1650	81,2	942.7	510	115.8
**LDH (U/L) (125–243)**		218	336	373	310	538		316	991	165	232	323	244
**D-dimer (ng/mL) (0–0.55)**			3.32	3.5	1.11	2.05		2.31	28.54	1.63	12.34	0.61	1.03
**IL 6 (pg/mL) (0–6)**			4.59	207.8	56.74	117.1	520		228	20.1	109.6		9.18

CRP: C-reactive protein; IL: interleukin; LDH: lactate dehydrogenase.

## RESULTS

### Case 1

**Figure 1:**
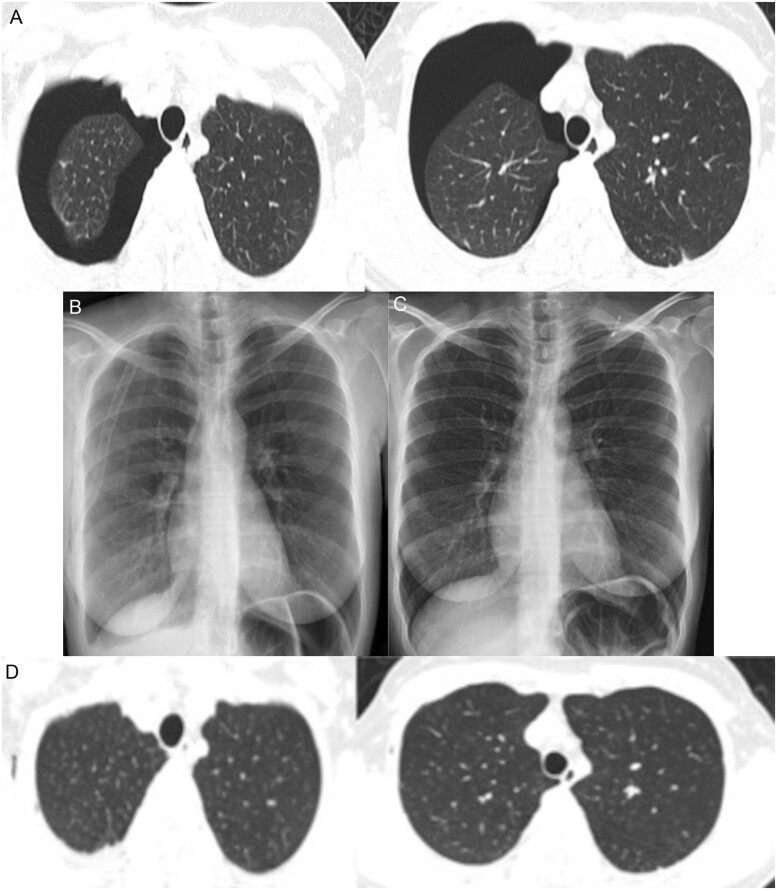
(**A**) Pneumothorax in the right lung on thorax computed tomography. No bulla or bleb was detected. (**B**) Lung expanded on X-ray image after chest tube thoracostomy. (**C**) Chest tube was removed on the fifth day of follow-up. (**D**) She was admitted to outpatient clinic 2 weeks after discharge, and her lungs observed expanded in both chest X-ray and thorax computed tomography.

A 40-year-old female patient with no known comorbidity was admitted to the emergency department with the complaints of a sore throat for 3 days and dyspnoea. Pneumothorax in the right lung and consolidation in the collapsed lung were observed on thorax computed tomography (TCT; Fig. 1A). No bullae or blebs were detected. Right-tube thoracostomy was applied to the patient. Her throat swab sample was COVID-19-positive. She recovered from the pneumothorax on the third day of follow-up (Fig. 1B), and the chest tube was removed on the fifth day of follow-up. The patient was discharged after the antiviral treatment was completed (Fig. 1C). The patient was admitted to an outpatient clinic 2 weeks after discharge, and her lungs were shown to be expanded in both chest X-ray and TCT (Fig. 1D). COVID-19 was not detected in a repeated nasopharyngeal swab test of the patient.

### Case 2

**Figure 2:**
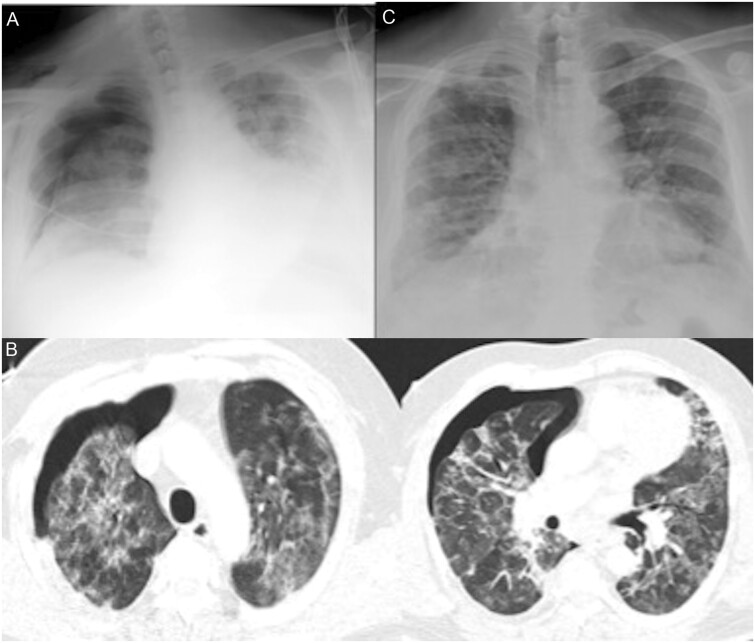
(**A**) Pneumothorax in the right lung was seen on chest radiography of the patient. (**B**) Thorax computed tomography of the patient revealed predominantly irregular ground-glass opacities in the peripheral zone and accompanying interstitial thickening in both lungs and pneumothorax on the right. (**C**) His lungs observed expanded in chest X-ray.

A 47-year-old male patient with the comorbidities of hypertension, coronary artery disease and type 1 diabetes mellitus was admitted to our hospital due to dyspnoea and palpitation. A nasopharyngeal swab sample from the patient was positive for COVID-19 RNA. The patient complained of sudden chest pain and shortness of breath on the second day of hospitalization. Pneumothorax in the right lung was seen on chest radiography of the patient (Fig. 2A). TCT of the patient revealed predominantly irregular ground-glass opacities in the peripheral zone, accompanying interstitial thickening in both lungs, and pneumothorax in the right lung (Fig. 2B). A right chest tube was applied to the patient. Atrial fibrillation was detected in an electrocardiogram taken of the patient due to the persistent of chest pain and shortness of breath. Pulmonary embolism was revealed on computed tomography angiography. Hypodense filling defects compatible with pulmonary thromboembolism allowing flow in pulmonary artery branches to the lower lobe of the right lung were observed. The patient received treatments for atrial fibrillation, pulmonary embolism and COVID-19. After 1 week of treatment, the polymerase chain reaction result was COVID-19- negative in a nasopharyngeal swab of the patient . Tube thoracostomy was terminated on the 6th day (Fig. 2C), and the patient was discharged on the 20th day of hospitalization.

### Case 3

A 75-year-old male patient with no comorbid condition was admitted to the hospital with complaints of dyspnoea, chest pain, cough and sputum. The patient had a history of hospitalization for 3 weeks, 1 week in the intensive care unit and 2 weeks in the ward, due to COVID-19 pneumonia 1 month previously. He had been discharged with an oxygen concentrator 1 week previously. Diffuse ground-glass opacities and interstitial thickening in the bilateral lung and cystic cavitary lesions in the left upper and lower lobes, and hydropneumothorax in the left hemithorax were seen in TCT. A chest tube was inserted into the left intrapleural space before the patient was admitted to the intensive care unit. The nasopharyngeal swab sample of the patient was negative for COVID-19 RNA. The pneumothorax was found to remain in a control chest radiograph of the patient taken on the first day. A second chest tube was inserted when it was observed that the pneumothorax had not recovered in a chest radiograph taken the next day. Although the pneumothorax was subsequently found to have healed in chest radiographs, worsening of the pulmonary infiltrates was observed. The patient developed sepsis during follow-up and died on the 11th day of hospitalization.

### Case 4

A 70-year-old female patient was admitted to the emergency department complaining of having suffered a cough and sudden shortness of breath for 4 days. Her medical history indicated that she received chemotherapy for breast cancer. A TCT scan showed diffuse ground-glass opacities and inter-septal thickening in the bilateral lung, and pneumothorax in the left hemithorax. A chest tube was inserted into the left intrapleural space. A nasopharyngeal swab sample from the patient was positive for COVID-19 RNA. Chest tube thoracostomy was terminated on the sixth day after the pneumothorax recovered. On the seventh day, the patient, whose general condition was stable and whose vital signs were normal, was discharged.

### Case 5

A 79-year-old male patient, diagnosed with COVID-19 in another hospital, was referred to us after computed tomography of the thorax showed deterioration in the ground-glass opacities observed in both lungs. Non-invasive mechanical ventilation (NIMV) was given in addition to medical treatment. The worsening of the lesions continued on the third day and the patient, who developed sudden respiratory distress due to decreased oxygen saturation on the fourth day, was intubated. Since the oxygen-saturation values did not improve after intubation, chest radiography was performed and a pneumothorax was found in the right hemithorax. The general condition of the patient deteriorated due to sepsis and he died on the sixth day from sepsis and respiratory failure.

### Case 6

An 86-year-old male patient was admitted to the emergency department with complaints of shortness of breath, fever and weakness. A TCT scan showed bilateral ground-glass opacities and hydropneumothorax in the right hemithorax. A right chest tube was inserted into the intrapleural space. A nasopharyngeal swab sample studied from the patient was positive for COVID-19 RNA. The medical history of the patient included coronary artery disease, chronic atrial fibrillation and hypertension. The patient, who received NIMV in addition to medical treatment, did not respond to the interventions. The patient died of acute respiratory distress syndrome (ARDS) after worsening pulmonary infiltrations on the second day.

### Case 7

A 76-year-old female patient had been hospitalized in our institution for 10 days due to COVID-19 pneumonia. Bilateral ground-glass opacities and a right pneumothorax were detected by TCT after sudden shortness of breath. A right chest tube was inserted. The pneumothorax recovered and the chest tube was terminated on the third day. The patient was on NIMV in combination with COVID-19-pneumonia treatment. She was discharged after 25 days of hospitalization due to accompanying liver and urinary system pathologies.

### Case 8

A 72-year-old male patient had previously been hospitalized for 1 week in an external hospital for COVID-19-induced pneumonia 45 days ago. He was admitted to our clinic after suffering a chest pain for 5 days. His medical history was uneventful. A TCT scan showed bilateral ground-glass opacities and a left pneumothorax. A left chest tube was inserted. The nasopharyngeal swab sample of the patient was negative for COVID-19 RNA. The chest tube was terminated on the fourth day and the patient was discharged on the fifth day.

### Case 9

An 88-year-old male patient was admitted to the emergency department with complaints of loss of appetite, weakness and general condition disorder that had continued for 15 days. His medical history included hypertension and DM. TCT showed diffuse microcystic changes on the left lung accompanying to a minimal pneumothorax, in addition to bilateral ground-glass opacities. As well as standard COVID-19 medical treatment, high-dose oxygen therapy was applied. The pneumothorax healed on the 4th day and the patient was discharged on the 13th day after the medical treatment was completed.

### Case 10

A 91-year-old male patient was admitted to the emergency department with complaints of dyspnoea. He was shown to have chronic obstructive pulmonary disease with bilateral diffuse emphysema and infiltrates with a minimal right anterior pneumothorax on TCT. High-dose oxygen therapy was applied. The pneumothorax healed after 2 days. The patient was discharged after recovery on the seventh day.

### Case 11

A 69-year-old male patient was admitted to the emergency room with sudden shortness of breath. Bilateral pulmonary infiltrates and a pneumothorax on the right lung were detected by TCT. Right chest tube thoracostomy was applied. The patient was discharged immediately after chest tube thoracostomy was terminated on the fifth day.

This study included only the patients with isolated SP detected radiological and, in this study, isolated SP was detected in all the cases and no pneumomediastinum was present (Table 1).

The main cause for hospitalization was pneumothorax, which was present in 7 (63%) of the 11 cases. In the same period (between March and December) over the 3 years prior to the COVID-19 pandemic, in total, 40 patients were hospitalized with a diagnosis of SSP (13, 12 and 15 cases, respectively). The annual number of SSP cases was found to have increased to 19 during the COVID-19 period, although this difference was not statistically significant (*P* = 0.267). Eleven of the 19 cases with SSP (57.89%) were associated with COVID-19 infection. No statistically significant difference was found between the tube thoracostomy treatment duration in the pre-COVID-19 period and that during the COVID-19 pandemic (*P* = 0.157; 6.23 and 5.8 days, respectively). Although the result was not statistically significant due to the limited number of patients, the risk of SSP showed a tendency to increase with COVID-19 infection (Table 1).

Our study evaluated cases of SP detected prior to NIMV and/or mechanical ventilation (MV). Patients who needed MV and/or NIMV after pneumothorax had a poor clinical course. These patients had a longer hospitalization period and higher morbidity and mortality compared to others (Table 1). Three of the 5 cases (60%) died while receiving MV (3 of the cases received NIMV, 1 received MV and 1 received both). MV was not required in 80% of the non-smoker cases (*n* = 4/5) and NIMV was applied in 20% (*n* = 1/5). Mortality was not observed in these cases. By contrast, MV/NIMV was applied in 66.6% of the smoker/ex-smoker cases (*n* = 4/6) and mortality was observed in 50% (*n* = 3). Although the clinical course of COVID-19 SP in smokers was worse and mortality was observed in 3 cases, there was no statistically significant difference in terms of the smoking and MV/NIMV relationship and the mortality and smoking relationship (*P* = 0.242, and *P* = 0.064, respectively). The small number of cases limited the statistical evaluation. The average pneumothorax recovery and tube thoracostomy termination time were 4.8 (2–11) days in smoker/ex-smoker cases and 4.75 (3–6) days in non-smoker cases. This difference was not statistically significant (*P* = 0.981; Table 1).

One notable finding of this study was the presence of lymphopaenia and elevated inflammatory markers including human C-reactive protein, lactate dehydrogenase, ferritin, D-dimer and interleukin-6 levels in most patients who developed SP (Table 2). Neutrophilia and lymphopaenia were detected in all cases except one (Case 4) who had received chemotherapy for breast cancer (Table 2).

## DISCUSSION

Pneumothorax is the accumulation of air in the space between the parietal pleura and the visceral pleura lining the lungs. A primary spontaneous pneumothorax can occur with no precipitating event, while an SSP is a complication of underlying lung disease. The mechanism of the injury, although not completely understood, may therefore be secondary to alveolar damage from the infection and a rupture of the alveolar wall due to increased pressure from the pronounced coughing that occurs in response to the virus [9]. In addition, an inflammatory reaction could also contribute to SP during lung infections. Some studies have reported that severe acute respiratory syndrome might independently result in cyst formation, even in the absence of MV, and that inflammatory exudates could play a role in this process [10, 11]. Pulmonary cystic lesions may develop in response to cellular fibromyxoid exudates, which form a valve in the bronchus [12]. Cytokine-storm syndrome is a critical clinical condition induced by a cascade of cytokine activation, which is characterized by overwhelming systemic inflammation, hyperferritinaemia, haemodynamic instability and multiple organ failure; this is now recognized as being a main cause of severe acute respiratory syndrome coronavirus 2 infection [13]. During the current COVID-19 pandemic, patients who have experienced respiratory failure have usually been subjected to COVID-19 protocols, while they were in emergency rooms including the possibility of positive-pressure ventilation, which can aggravate the clinical course of a pneumothorax.

Since the first cases of COVID-19 were described, pneumothorax has been characterized as a potential, though uncommon, complication. Chen *et al.* [3] described only one patient with SP out of 99 confirmed COVID-19 cases with a pneumothorax. Yang *et al.* [6], in an autopsy study consisting of 92 patients, found just one case with the same diagnosis. Salehi *et al.* [14] reviewed computed tomography findings and determined that pneumothorax was uncommon. To date, 1855 COVID-19 patients have been treated in our hospital. In total, 1498 of these cases were followed up in the ward and 357 were followed up in the intensive care unit. The first case of pneumothorax was detected incidentally, and no bullae or other pulmonary parenchymal lesions or pulmonary infiltration were detected radiologically. Pneumonic infiltrates were present in all other cases. In addition, pleural effusion and a cystic cavitary lesion were found in one case, pleural effusion was identified in another and microcystic changes were detected in an additional patient.

Data from autopsy studies of COVID-19 patients have shown that the predominant pulmonary pathological findings are diffuse alveolar damage, similar to in severe acute respiratory syndrome patients, and the development of a pneumothorax, which supports the pulmonary parenchymal damage hypothesis [15, 16]. Other findings have included acute bronchopneumonia and pulmonary embolism [15, 16]. Ten of our 11 cases had bilateral ground-glass opacities or pulmonary infiltrates in the parenchyma and were thus treated as COVID-19 pneumonia.

Late sequelae due to COVID-19 pneumonia have been reported and can cause pneumothorax [17]. In our study, Cases 3 and 8 had been hospitalized for COVID-19 pneumonia about 1 month earlier, treated for COVID-19, discharged and then subsequently re-admitted to the emergency department due to pneumothorax. Case 3 was followed up in the intensive care unit due to progressive COVID-19 pneumonia and this patient died because of ARDS. Case 8 did not require any additional treatment other than tube thoracostomy and this patient was discharged after 3 days with full recovery. In our opinion, pneumothorax could be diagnosed as a late sequela complication of COVID-19 pneumonia in these cases.

COVID-19 induced pneumothorax and pneumomediastinum have been commonly reported together in the literature [9, 18]. All of our cases in this study had isolated SP and 2 among those had pleural effusion accompanying to SP. We attributed the cause of pleural effusion to the length of time between the symptoms developing and hospitalization. There was no pneumomediastinum detected radiologically in our cases. Unlike most of the literature, non-spontaneous (e.g. barotrauma-induced) pneumothorax cases were not included in this study. The first case was detected incidentally without any pulmonary pathology, while the other 10 cases were defined as SSP due to COVID-19 pneumonia.

**Figure 3:**
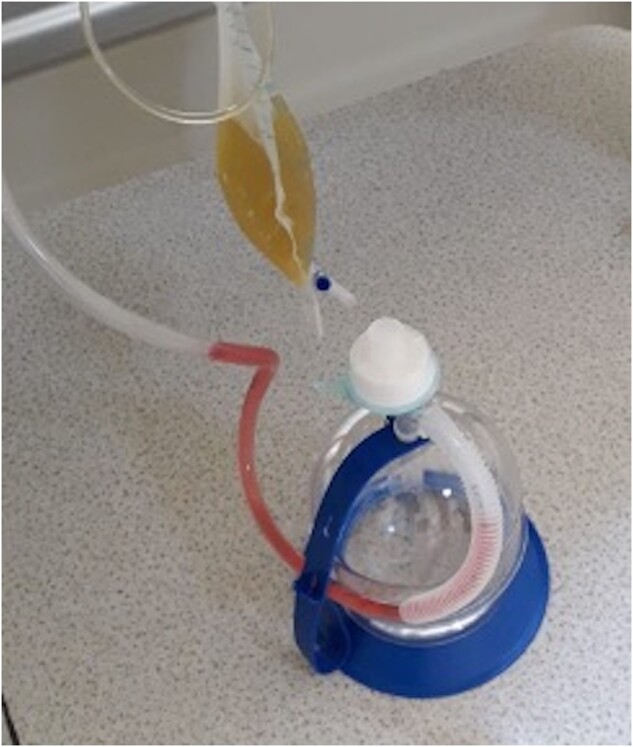
A filter was used in the pulmonary drainage system.

It has previously been suggested that the development of pneumothorax during coronavirus infection is an important prognostic marker [8, 19]. However, COVID-19 treatment in patients with pneumothorax may lead to additional comorbidities and complications. Notably, chest-drain insertion for pneumothorax could be considered as an aerosol-generating procedure, and severe acute respiratory syndrome coronavirus 2 viral RNA has recently been detected in the pleural fluid at post-mortem [20, 21]. It is essential that clinicians are provided with appropriate personal protective equipment when performing aerosol-generating procedures such as chest-drain insertion, and that droplet-minimizing modifications are implemented, including digital drainage systems, connecting the drainage circuit to a wall suction line and using filters to limit viral spread [22, 23]. A filter was used in the pulmonary drainage system in all 9 of our cases (Fig. 3). After understanding the mechanism of potential COVID-19 transmission during pneumothorax treatment, it is imperative to develop preventive approaches of necessary interventions.

Lymphopaenia and elevated inflammatory markers, including C-reactive protein, lactate dehydrogenase, ferritin, D-dimer and interleukin-6 levels, were found in most of the patients who developed SP in the present study (Table 2). This was consistent with recently published studies that have examined the possible mechanisms of COVID-19-induced lung injury. Cytokine storms have been thought to play a role in the pathophysiology of the disease. This type of hyperactive and dysregulated immune response may lead to a hyperinflammatory form of ARDS and is associated with critical illness and increased mortality [24, 25]. Furthermore, thrombosis and microangiopathy were observed in lung tissue obtained from patients with ARDS who had died from COVID-19 viral infection. This is thought to play a role in lung injury, although further studies are needed to examine the implications of these findings [26]. In our institution, all patients with COVID-19 infection were placed on prophylactic or therapeutic anticoagulation, unless contraindicated. The choice and dose of the anticoagulants were based on a multidisciplinary discussion that included the haematology specialists.

Neutrophilia and lymphopaenia can cause acute lung injury, leading to pneumothorax [27]. Neutrophilia and lymphopaenia were detected in all our cases except one (Case 4), who had received chemotherapy for breast cancer. These prognostic factors exacerbated pneumothorax due to COVID-19 and may increase the length of hospital stay, thus escalating the progression of infection parameters and related morbidity–mortality.

Delay in admission to hospital and severe pneumonia progression was detected in 3 patients who died due to sepsis, ARDS and multi-organ failure. Chest tube thoracostomy was sufficient in the treatment of pneumothorax in 9 cases, whereas high-dose oxygen therapy was sufficient in 2 cases. The lungs were expanded in all our cases and surgery was not required.

The risk of pneumothorax was increased in COVID-19 patients treated with NIMV and MV [28]. Our study evaluated cases of SP detected prior to NIMV and MV. Patients who needed MV and NIMV after pneumothorax had a poor clinical course, longer hospitalization period and higher morbidity and mortality compared to others (Table 1). Three of the 5 cases (60%) died while undergoing MV (3 received NIMV, 1 received MV and 1 received both).

Smoking is a poor prognostic factor in COVID-19 infection and pneumothorax. Mallick *et al.* [29] reported on a smoking case in which a poor clinical course was observed and the patient died. In our study, MV/NIMV was applied in 66.6% of the smoker/ex-smoker cases and mortality was observed in 50%. Although the clinical course of COVID-19 patients with SP in smokers was worse, and mortality was observed in 3 cases, there was no statistically significant difference in terms of smoking and MV/NIMV relationship and mortality and smoking.

The rate of hospitalization has been reported to increase with the incidence of SP due to COVID-19 [30]. The main cause for hospitalization in 7 of our 11 cases was pneumothorax. In the same period over the 3 years prior to the COVID-19 pandemic, in total, 40 patients were hospitalized with a diagnosis of SSP. The annual number of the SSP cases was found to have increased, although the difference was not statistically significant. No statistically significant difference was found between the tube thoracostomy treatment duration in the pre-COVID-19 period and that during the pandemic.

In this study, compared to the previous years, the coexistence of COVID-19 infection with pneumothorax increased the number of SP cases and the rate of hospitalization and length of stay. Pneumothorax can therefore be considered as a poor prognostic factor in COVID-19 infection.

Inflammation is common in COVID-19 pneumonia. It is possible that the developing inflammation poses a risk for SP and increases the incidence of pneumothorax. In such cases, early anti-inflammatory treatment, such as corticosteroid therapy, might decrease the frequency. In addition, during the follow-up of patients with COVID-19, new symptoms and findings such as novel chest pain, onset of shortness of breath, increase in shortness of breath or deepening of hypoxaemia should be considered as warning signs for pneumothorax.

## CONCLUSION

The increased number of cases of pneumothorax suggests that it may be a complication of COVID-19 infection. During medical treatment for COVID-19, pneumothorax may be the only reason for hospitalization. Although tube thoracostomy is a sufficient treatment option in most cases, clinicians should be aware of the difficulties that may arise in diagnosis and treatment.

**Conflict of interest:** none declared.

## Author contributions

**Hakki Ulutas:** Data curation; Formal analysis; Project administration; Resources; Writing – original draft; Writing—review & editing). **Muhammet Reha Celik:** (Resources; Writing—original draft; Writing—review & editing). **Ilham Gulcek:** Data curation; Formal analysis; Project administration; Resources. **Muhammed Kalkan:** Investigation; Visualization. **Mehmet Ağar:** Data curation; Investigation; Visualization; Writing—review & editing. **Talat Kılıç:** Data curation; Methodology; Supervision; Writing—original draft; Writing—review & editing. **Emine Gulcek:** Investigation; Resources; Writing—original draft.

## Reviewer information

Interactive CardioVascular and Thoracic Surgery thanks Vanessa Diaz-Ravetllat, Katrin Welcker, Federico Tacconi and the other anonymous reviewers for their contribution to the peer review process of this article.

## ABBREVIATIONS

ARDS: Acute respiratory distress syndrome
COVID-19: Coronavirus diseases 2019
MV: Mechanical ventilation
NIMV: Non-invasive mechanical ventilation
SP: Spontaneous pneumothorax
SSP: Secondary spontaneous pneumothorax
TCT: Thorax computed tomography
